# Copy number loss of *KDM5D* may be a predictive biomarker for ATR inhibitor treatment in male patients with pulmonary squamous cell carcinoma

**DOI:** 10.1002/cjp2.350

**Published:** 2023-11-16

**Authors:** Ayako Ura, Takuo Hayashi, Kazumasa Komura, Masaki Hosoya, Kazuya Takamochi, Eiichi Sato, Satomi Saito, Susumu Wakai, Takafumi Handa, Tsuyoshi Saito, Shunsuke Kato, Kenji Suzuki, Takashi Yao, the Tokyo Metropolitan Innovative Oncology Research Group (TMIG)

**Affiliations:** ^1^ Department of Human Pathology Juntendo University Graduate School of Medicine Tokyo Japan; ^2^ Department of Urology Osaka Medical and Pharmaceutical University Osaka Japan; ^3^ Translational Research Program Osaka Medical and Pharmaceutical University Osaka Japan; ^4^ Department of Clinical Oncology Juntendo University Graduate School of Medicine Tokyo Japan; ^5^ Department of General Thoracic Surgery Juntendo University Graduate School of Medicine Tokyo Japan; ^6^ Department of Pathology Institute of Medical Science (Medical Research Center), Tokyo Medical University Tokyo Japan; ^7^ Division of Clinical Laboratory National Center for Global Health and Medicine Tokyo Japan

**Keywords:** ataxia‐telangiectasia and Rad3‐related kinase, DNA damage response, *KDM5D*, squamous cell carcinoma

## Abstract

A limited number of patients with lung squamous cell carcinoma (SCC) benefit clinically from molecular targeted drugs because of a lack of targetable driver alterations. We aimed to understand the prevalence and clinical significance of lysine‐specific demethylase 5D (*KDM5D*) copy number loss in SCC and explore its potential as a predictive biomarker for ataxia‐telangiectasia and Rad3‐related (ATR) inhibitor treatment. We evaluated *KDM5D* copy number loss in 173 surgically resected SCCs from male patients using fluorescence *in situ* hybridization. *KDM5D* copy number loss was detected in 75 of the 173 patients (43%). Genome‐wide expression profiles of the transcription start sites (TSSs) were obtained from 17 SCCs, for which the cap analysis of gene expression assay was performed, revealing that upregulated genes in tumors with the *KDM5D* copy number loss are associated with ‘cell cycle’, whereas downregulated genes in tumors with *KDM5D* copy number loss were associated with ‘immune response’. Clinicopathologically, SCCs with *KDM5D* copy number loss were associated with late pathological stage (*p* = 0.0085) and high stromal content (*p* = 0.0254). Multiplexed fluorescent immunohistochemistry showed that the number of tumor‐infiltrating CD8^+^/T‐bet^+^ T cells was lower in SCCs with *KDM5D* copy number loss than in wild‐type tumors. In conclusion, approximately 40% of the male patients with SCC exhibited *KDM5D* copy number loss. Tumors in patients who show this distinct phenotype can be ‘cold tumors’, which are characterized by the paucity of tumor T‐cell infiltration and usually do not respond to immunotherapy. Thus, they may be candidates for trials with ATR inhibitors.

## Introduction

Lung squamous cell carcinoma (SCC), which accounts for approximately 25–30% of non‐small cell lung cancers (NSCLCs) carries a high rate of mutations and is found mostly among smokers [1]; frequently altered genes include *TP53*, *PIK3CA*, *CDKN2A*, *SOX2*, and *CCND1* [2, 3, 4]. In contrast to lung adenocarcinoma, which has multiple targetable alterations such as mutant *EGFR*, *BRAF*, *ERBB2*, and *KRAS*; rearranged *ALK*, *ROS1*, *RET*, and *NTRK1*; and *MET* exon 14 skipping [5, 6, 7], patients with SCC do not clinically benefit from molecular targeted drugs. Thus, identifying targetable alterations and finding those of clinical relevance is an unmet need for patients with SCC.

In cancers that lack targetable molecular alterations, targeting the well‐defined phenotypic hallmarks could have therapeutic benefits [8]. One such hallmark is genomic instability, which promotes genetic diversity and thereby drives the acquisition of multiple hallmark capabilities. DNA damage resulting from unabated replication (i.e. DNA replication stress) is a major source of genomic instability in cancers and precancerous lesions [9, 10, 11]. Cellular response to DNA damage is mainly regulated by the ataxia‐telangiectasia mutated‐checkpoint kinase 2 and ataxia‐telangiectasia and Rad3‐related (ATR)‐checkpoint kinase 1 pathways [12]. Pharmacological inhibition of ATR exacerbates cell death and enhances antitumor activity of inhibitors of DNA topoisomerase I, a nuclear enzyme that suppresses genomic instability [13, 14]. Indeed, prostate adenocarcinoma cells with DNA replication stress induced by the loss of expression of male‐specific histone‐demethylase lysine demethylase 5D (*KDM5D*) are sensitive to an ATR inhibitor [15]. This observation indicates that the loss of expression of *KDM5D* could serve as a biomarker to predict the efficacy of ATR inhibition, which may help select patients eligible for this new targeted therapy [16]. *KDM5D*, located on the Y chromosome, is capable of demethylating the active transcriptional markers H3K4me2 and H3K4me3 [17]. Despite extensive genome‐wide analysis using next‐generation sequencing, copy number alteration of genes on the Y chromosome has not been well studied.

Although ATR inhibitors have not yet been approved by the US Food and Drug Administration for lung cancer treatment, a phase II trial of an ATR inhibitor (berzosertib) in combination with a highly selective topoisomerase I inhibitor (topotecan) in patients with relapsed small cell lung cancer (SCLC), all of whom had evidence of disease progression after conventional treatment including chemotherapy or immunotherapy before participation, showed prolonged survival [18]. Furthermore, preliminary signs of clinical efficacy of berzosertib in combination with gemcitabine or another ATR inhibitor, ceralasertib, in combination with carboplatin were observed in a small number of patients with advanced tumors including NSCLC, suggesting an early proof of concept for this drug class; however, these trials suggested that further clinical trials may be best undertaken in a patient population selected for potential biomarkers [19, 20, 21].

Here, we analyzed the ramifications of *KDM5D* copy number loss in SCC using molecular and clinicopathologic approaches. Histologically, we focused on cellular findings correlated with worse clinical outcomes in SCC, including nuclear size and tumor budding [22], and stromal findings to clarify clinicopathological characteristics of patients with *KDM5D* copy number loss. We aimed to investigate whether such loss is a suitable predictive biomarker for ATR inhibitor treatment in male patients with SCC in further clinical trials.

## Materials and methods

### Study population

We screened the archives of the Department of Human Pathology, Juntendo University School of Medicine, Tokyo, Japan to identify patients who underwent complete lung tumor resection between January 2008 and January 2014. We obtained data on the following clinicopathological parameters: age, sex, smoking status, tumor size, lymphovascular invasion, lymph node and distant metastases, resection type, and mutation status of *EGFR* and *KRAS*. All tumors were resected at the Department of General Thoracic Surgery of Juntendo University Hospital. Pathological diagnoses were based on the 2021 WHO classification [23]. To clarify clinicopathological features of SCC with *KDM5D* copy number loss, we excluded female patients in the present study because *KDM5D* is located on the Y chromosome so, theoretically, no female patients with SCC express *KDM5D*. The archives contained data from 194 male patients with SCCs. All patients were followed‐up via regular physical and blood examinations, with mandatory radiography, computed tomography, or magnetic resonance imaging. Additionally, 65 male patients with SCLC who underwent surgical resection at Juntendo University Hospital between 2008 and 2020 were assigned to the control group to confirm the association of copy number loss of *KDM5D*, which was detected using fluorescence *in situ* hybridization (FISH) and droplet digital PCR (ddPCR). In both groups, all tissues were fixed in 10% buffered formalin and embedded in FFPE after routine processing, and these tumors were assembled into tissue microarrays (TMAs), using 1.5–2.0‐mm cores sampled from one or two different representative areas of each FFPE tissue block. The study was performed in accordance with the Declaration of Helsinki. The patients were not required to provide consent for participation in the study, but we offered them an opt‐out option, which allows patients to express a choice to object to their confidential patient information being used in this research. Individuals cannot be identified from the data presented herein. This study was approved by the Institutional Review Board of Juntendo University (Approval No. 2019123).

### Histopathologic examination

We evaluated tumor budding, nuclear size, stromal content, and spread through alveolar spaces using representative slides of SCCs. Tumor budding was assessed in areas showing the most extensive budding activity. Tumor budding was defined as a tumor nest composed of less than five tumor cells. Tumor stromal content was categorized as low (<50% of the entire tumor area) and high (≥50%). The presence of spread through alveolar spaces was defined as tumor cell nests or single cells spreading within air spaces beyond the edge of the main tumor. Immunohistochemical examination was performed on TMAs of SCCs using antibodies against p40 (BC28; Biocare, Copenhagen, Denmark) and programmed death‐ligand 1 (PD‐L1; clone 22C3; DAKO/Agilent Technologies, Glostrup, Denmark), following the manufacturers' recommendations. Tumors were considered PD‐L1 positive if ≥1% of the tumor cells were stained, i.e. tumor proportion score ≥1%. *In situ* hybridization for Epstein–Barr virus‐encoded RNA (Roche, Tucson, AZ, USA) was performed on TMAs of SCCs, following the manufacturer's recommendations.

### Digital assessment approach for nuclear size in SCC

For measuring nuclear area, TMA images which were archived as high‐resolution whole‐slide images were analyzed in QuPath (Version 0.4.3), an open‐source software for digital image analysis [24]. We analyzed the nuclear area of tumor cells, with a minimum of 100 cells, detecting and labeling with the QuPath application ‘cell detection’ and ‘detection measurement’ in each TMA core. We used QuPath's polygon tool for annotation of areas of interest after detecting TMA cores. After visually comparing different settings of the parameter for optimal detection of tumor cells, we used the default or proper settings for each case (supplementary material, Figure S1A). Overlapping nuclei that could not be measured were omitted. We also analyzed nuclear area of lymphocytes by selecting five TMA cores and averaging them as a control (supplementary material, Figure S1B).

### FISH

FISH analysis of the TMAs was performed using a two‐color KDM5D/X chromosome (CenX) probe mix, which was provided by the Molecular Cytogenesis Core at Memorial Sloan Kettering Cancer Center (New York, USA). From the 194 male patients with SCCs, TMAs of 173 tumors were available for FISH analysis. The probe mix consisted of a bacterial artificial chromosome clone containing full‐length *KDM5D* (Yq11) (RP11‐188C1 and RP11‐204P21; labeled with red dUTP) and a centromeric repeat plasmid specific to CenX (pSV2x5; labeled with green dUTP). Probe labeling, tissue processing, hybridization, post‐hybridization washing, and fluorescence detection were performed according to standard laboratory procedures [15]. Images were obtained with an all‐in‐one fluorescence microscope (BZ‐X800; KEYENCE, Osaka, Japan) equipped with a Plan Apochromat ×40 objective (NA0.95, BZ PA40; KEYENCE). Red fluorescence was detected using a TexasRed filter (ex: 560/40 nm, em: 630/75 nm, dichroic: 585 nm, OP‐87765; KEYENCE) and green fluorescence was detected using a GFP filter (ex: 470/40 nm, em: 525/50 nm, dichroic: 495 nm, OP‐87763; KEYENCE). A minimum of 50–300 nuclei per case were evaluated. Copy number loss of *KDM5D* was defined as cases in which more than 90% of tumor cells showed absence of a red (*KDM5D*) signal, as previously described [15].

### DNA extraction and digital droplet PCR

Genomic DNA was extracted from FFPE of SCLC tissues using the QIAamp DNA FFPE Tissue Kit (Qiagen, Venlo, Netherlands). Primers and probes used in ddPCR were as follows: PrimePCR™ ddPCR™ Expression Probe Assay, *KDM5D* (FAM probe, Human, Catalog ID: 10031252, Unique Assay ID: dHsaCPE50322209; Bio‐Rad Laboratories, Hercules, CA, USA) and PrimePCR™ ddPCR™ Copy Number Assay, *SPIN4* (HEX probe, Human, Catalog ID: 10031243, Unique Assay ID: dHsaCP2506729; Bio‐Rad Laboratories). Probes were designed on *KDM5D* locus for FAM reporters and reference sites for HEX reporters (*SPIN4*: X chromosome). The QX200 system (Bio‐Rad Laboratories) was used in the study. The reactions were performed with 20‐μl mixtures of 10 μl of 2× ddPCR Supermix for probes (no dUTP) (Bio‐Rad Laboratories), primers (900 nmol/l), probes (250 nmol/l), and 50 ng of the genomic DNA sample. The QX200 droplet generator (Bio‐Rad Laboratories) converted each reaction mix to droplets. Droplet‐partitioned samples were then transferred to a 96‐well plate, sealed, and cycled in a C1000 Touch thermal cycler (Bio‐Rad Laboratories) under the following cycling protocol: 95 °C for 10 min, followed by 40 cycles at 94 °C for 30 s, 60 °C for 60 s, and a 10‐min incubation at 98 °C. The products were then read on the QX200 droplet reader (Bio‐Rad Laboratories). At least two negative control wells with no genomic DNA template were included in each run. The data analysis was conducted with QuantaSoft droplet reader software v1.7.4 (Bio‐Rad Laboratories). *KDM5D*/*SPIN4* ratio <0.1 was used as an indicator of copy number loss of *KDM5D*.

### Multiplexed fluorescent immunohistochemistry

Multiplexed fluorescent immunohistochemistry was performed using the tyramide signal amplification method with an Opal IHC kit (Akoya Biosciences, Marlborough, MA, USA) according to the manufacturer's instructions. Tissue sections, of thickness 4 μm, were cut from FFPE tumor specimens and then baked at 60 °C onto adhesive glass slides for 30 min before deparaffinization. The primary antibodies used were anti‐human CD8 (clone C8/144b; DAKO), anti‐human T‐bet (Santa Cruz Biotechnology, Dallas, TX, USA), and cytokeratin (clone AE1/AE3; DAKO). A high‐pH target‐retrieval solution (DAKO) was used for antigen retrieval, and immunoactive buffer (Matsunami Glass, Osaka, Japan) was used for antibody stripping. Opal 520, 540, and 650 were used for labeling CD8, T‐bet, and cytokeratin, respectively. A horseradish peroxidase‐labeled secondary detection system (EnVision Plus, DAKO) was used as a catalyst for fluorophore‐conjugated tyramide. Heating at 95 °C for 20 min was performed for primary antigen unmasking and antibody stripping after each fluorescent labeling.

### Image analysis and quantification

Multiplexed fluorescent‐labeled images of three randomly selected fields (669 μm × 500 μm each) were captured with an automated imaging system (Vectra ver. 3.0; Akoya Biosciences). An image analyzing software program (InForm; Akoya Biosciences) was used to segment cancer tissue into cancer cell nests (intratumoral) and the framework (stromal) region, and to detect immune cells with CD8 and T‐bet expression. Training sessions for tissue segmentation and phenotype recognition were repeated until the algorithm reached the level of confidence recommended by the program supplier (at least 90% accuracy) before performing the final evaluation.

### Bioinformatic analysis of the cap analysis of gene expression data

Quantitative transcription start site (TSS)‐level expression profiles were obtained from the 17 SCCs, for which the cap analysis of gene expression (CAGE) assay was performed as previously described [25]. In brief, the CAGE reads were aligned to the reference genome (hg19) with a high mapping quality of ≥20. The aligned CAGE reads were counted in each region of the FANTOM5 robust peaks [26], a reference set of TSS regions, as raw signals for promoter activity. The expression levels of individual TSSs were quantified as counts per million. Inactive TSS regions, with counts per million ≤1 in more than 77% of samples, were filtered out [27]. A differential expression analysis was performed using R (version 4.2.1; https://www.r-project.org/; R Foundation for Statistical Computing, Vienna, Austria) with the edgeR package [28]. Differentially expressed TSSs were defined as fold change cutoff >2 and false discovery rate <0.05. To characterize genes that were correlated with *KDM5D* copy number loss that was detected using FISH, gene set enrichment analysis was performed using the *g:Profiler* website [29], which includes gene set databases such as Gene Ontology [29, 30], TRANSFAC [30], and The Human Protein Atlas [31]. The distances between the samples for the selected genes were calculated in terms of Euclidean distances for counts per million, and average linkage clustering was performed.

### Statistical analysis

The correlations between the results of ddPCR and FISH were assessed using Spearman's rank correlation. Categorical variables were analyzed using Fisher's exact or chi‐square test. To determine prognostic significance, we performed Kaplan–Meier survival analysis. These statistical analyses were performed using GraphPad Prism® version 9.0 (GraphPad Software, San Diego, CA, USA). Results with *p* < 0.05 were considered statistically significant. Associations between *KDM5D* and other genes were assessed using Spearman's rank correlation. A series of statistical analyses were performed in R with the edgeR package [28].

## Results

### Clinicopathological characteristics of the study cohort

**Figure 1:**
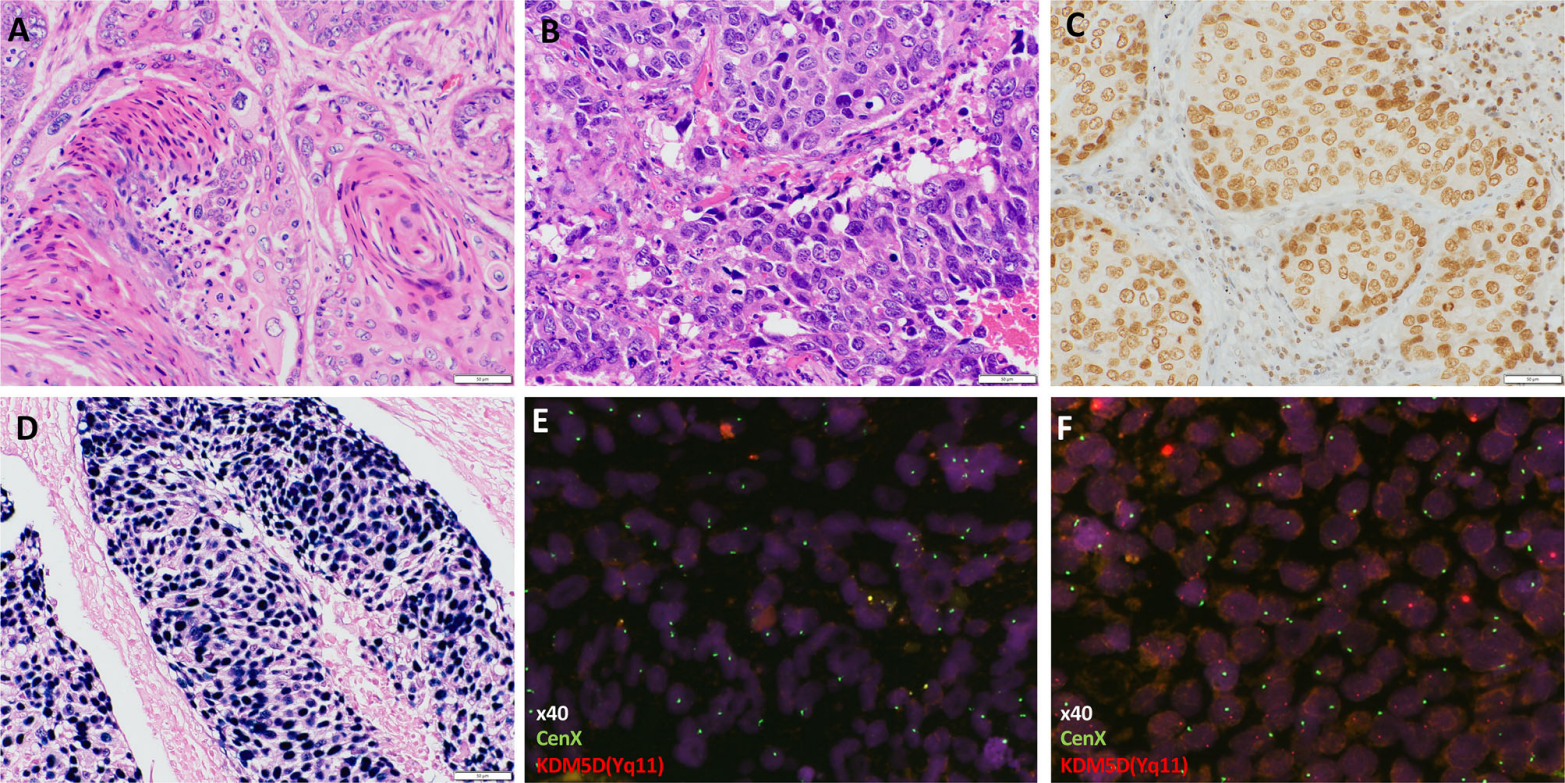
Pathological findings of SCC. Representative hematoxylin and eosin staining of (A) well‐differentiated and (B) poorly differentiated SCC. (C) Representative image of diffuse immunohistochemical staining of p40 in a poorly differentiated SCC. (D) A case of lymphoepithelial carcinoma was Epstein–Barr virus‐encoded RNA positive, as determined using *in situ* hybridization. (E and F) FISH analysis using a two‐color lysine‐specific demethylase 5D (KDM5D) (Yq11/red)/CenX (green) probe mix. Representative images showing (E) loss of *KDM5D* in a SCC and (F) *KDM5D*‐positive SCC.

The clinicopathological characteristics of the 173 male patients with SCC whose *KDM5D* copy number was examined are shown in Table 1. The median age of the patients with SCC was 70.5 years; 159 patients (91.9%) were heavy smokers (Brinkman index >400) and the remaining 14 patients were never or light smokers (Brinkman index ≤400). Histologically, 117 (67.6%) and 56 (32.4%) cases were keratinizing and nonkeratinizing type SCCs, respectively (Figure 1A,B). Immunohistochemically, no tumor expressed TTF‐1, whereas all tumors exhibited diffuse positivity for p40 (Figure 1C). Epstein–Barr virus‐encoded RNA was positive in a patient with nonkeratinizing type SCC, which led to the pathological diagnosis of lymphoepithelial carcinoma (Figure 1D). Furthermore, 71 (40%), 58 (33%), and 44 (25%) tumors had no, low, and high expression of PD‐L1, respectively. Genetically, five tumors (2.9%) harbored *KRAS* mutations. Among five patients with SCCs harboring *KRAS* mutations, three, one, and one were G12V, G12D, and G13C, respectively.

**Table 1:** Clinicopathological characteristics of male patients with lung squamous cell carcinoma

Characteristics	All patients, *n* = 173	*KDM5D* loss (+), *n* = 75	*KDM5D* loss (−), *n* = 98	*p* value
Age (range)	69.71 (40–90)	69.17 (43–88)	70.14 (40–90)	0.4358
Smoking level				
≤400	14	5	9	0.5885
>400	159	70	89	
Size				
<20 mm	25	8	17	0.2768
≥20 mm	148	67	81	
Tumor stage				
pT1	41	16	25	0.4759
pT2‐4	129	59	70	
Nodal status				
N0	102	37	65	0.0107
N1/N2/N3	65	37	28	
TNM stage				
I	78	26	52	0.0085
II–IV	91	49	42	
Lymphatic invasion				
Absent	123	50	73	0.3106
Present	50	25	25	
Vessel invasion				
Absent	62	23	39	0.2632
Present	111	52	59	
*EGFR* mutation				
Absent	173	75	98	>0.9999
Present	0	0	0	
*KRAS* mutation				
Absent	168	73	95	>0.9999
Present	5	2	3	
PD‐L1 (22C3) immunoreactivity				
No expression	71	29	42	0.3809
Low expression	58	23	35	
High expression	44	23	21	
Histological type				
Keratinizing type	117	51	66	>0.9999
Nonkeratinizing type	56	24	32	
Budding (per HPF)				
Present	78	38	40	0.2711
Absent	87	34	53	
Smallest tumor cell nest (overall)				
0–10	85	38	47	0.7557
>10	81	34	47	
Nuclear area (μm^2^)				
Median (range)	54.28 (26.30–120.50)	53.65 (26.30–92.67)	56.20 (27.74–120.50)	0.1064
Stromal content				
High (≧50%)	66	35	31	0.0254
Low (<50%)	100	37	63	
STAS				
Present	74	29	45	0.1887
Absent	76	38	38	

HPF, high power field; STAS, spread through alveolar spaces.

### Copy number loss of *KDM5D* in lung carcinomas

First, as a control, copy number loss of *KDM5D* was evaluated in SCLCs using ddPCR, a method that can absolutely quantify the *KDM5D* copy number without the need for standard curves. In SCLC, the median levels of *KDM5D* and *SPIN4* were 608 and 2256 (copies/μl), respectively (supplementary material, Table S1). Copy number loss of *KDM5D* (*KDM5D/SPIN4* ratio <0.1) was identified in 10 tumors (15%) using ddPCR, and there was correlation between the results of the copy number loss of *KDM5D* by ddPCR and FISH (*p <* 0.0001) (supplementary material, Figure S2). We then determined the frequency of copy number loss of *KDM5D* in TMA samples of SCCs using FISH (Figure 1E,F). Among the 173 patients with SCC, *KDM5D* copy number loss was detected in 75 SCCs (43%).

### CAGE data analyses reveal that *KDM5D* copy number loss correlates with cell cycle in SCC

**Figure 2:**
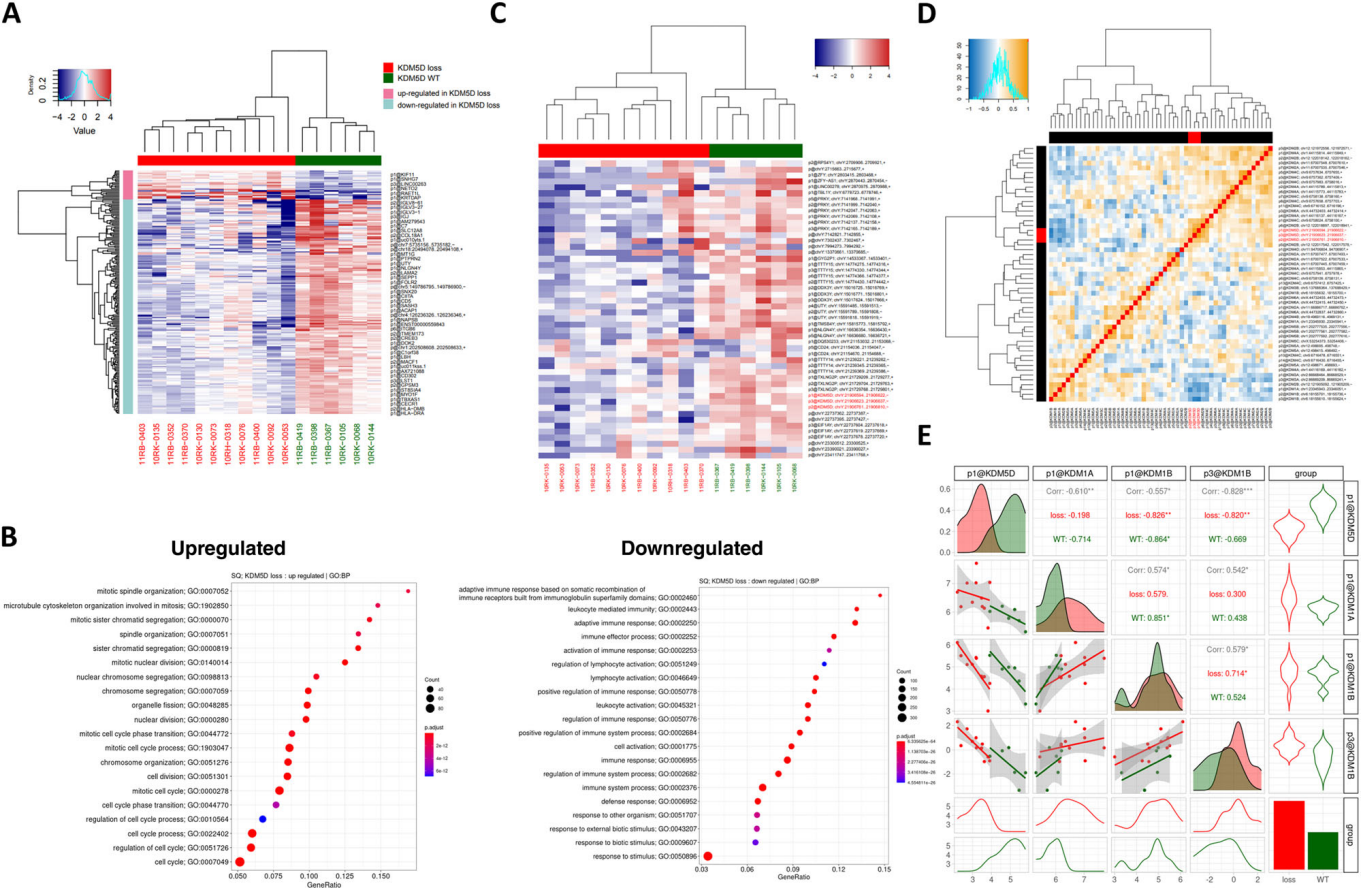
TSS‐level expression of *KDM5D* in SCC. (A) Two distinct clusters were observed in clustering based on the *KDM5D* copy number status. Red and green indicate loss of *KDM5D* and wild‐type tumors, respectively. (B) Top 20 terms of gene set enrichment analysis, performed using g:Profiler. The upregulated genes in SCC with copy number loss of *KDM5D* were associated with the term ‘cell cycle’, whereas downregulated genes in SCC with copy number loss of *KDM5D* were associated with ‘immune response’. (C) TSS‐level expression profiles of the genes on the Y chromosome. Genes are listed in a linear order on the chromosome. The expression of TSSs of genes on the Y chromosome was lower in tumors with *KDM5D* copy number loss (red) than in wild‐type tumors (green). (D) Hierarchical clustering of Spearman's rank correlation among all TSSs of *KDM* family genes. (E) Scatter plots and Spearman's rank correlation of TSSs of *KDM5D*, *KDM1A*, and *KDM1B*. Note the inverse correlations between the expression of TSS region 1 of *KDM1A* (*r* = −0.610) and *KDM1*B (*r* = −0.557), and TSS region 3 of *KDM1B* (*r* = −0.828).

To further explore the mechanism by which copy number loss of *KDM5D* contributes to the phenotype in SCC, we performed bioinformatic analysis of comprehensive transcriptome data using the CAGE sequence. In total, 44,529 TSSs were identified (supplementary material, Table S2). First, we compared the expression of TSS region 1 of *KDM5D* in tumors with and without *KDM5D* copy number loss. The results indicated that the expression of TSS region 1 of *KDM5D* was significantly lower in tumors with *KDM5D* copy number loss than in wild‐type tumors (*p* < 0.0001). Next, we comprehensively investigated the TSSs of whole genes that were positively or negatively correlated in tumors with *KDM5D* copy number loss. In the datasets, 208 TSSs were differentially expressed between SCCs with and without *KDM5D* copy number loss (Figure 2A and supplementary material, Table S3). Gene set enrichment analysis revealed that upregulated genes in tumors with the *KDM5D* copy number loss are associated with the cell cycle terms ‘mitotic spindle organization’, ‘microtubule cytoskeleton organization involved in mitosis’, and ‘nuclear division’, which is consistent with the finding that the loss of *KDM5D* expression is associated with acceleration of the cell cycle and mitotic entry [15]. On the contrary, downregulated genes in tumors with *KDM5D* copy number loss were associated with the immune response terms ‘adaptive immune response’, ‘immune effector process’, and ‘activation of immune response’ (Figure 2B).

We further evaluated the TSS‐level expression profiles of the genes on the Y chromosome. The analysis involved 40 TSSs of 18 genes, including *KDM5D*. Overall, the expression of genes on the Y chromosome, especially around *KDM5D* including *TXLNG2P* and *TTTY14*, was significantly lower in tumors with *KDM5D* copy number loss than in wild‐type tumors (Figure 2C). Lastly, we evaluated the TSS‐level expression profiles of KDMs in tumors with and without *KDM5D* copy number loss. In addition to *KDM5D*, 50 TSSs from 14 genes were identified, namely, *KDM1A*, *KDM1B*, *KDM2A*, *KDM2B*, *KDM3A*, *KDM3B*, *KDM4A*, *KDM4B*, *KDM4C*, *KDM4D*, *KDM5A*, *KDM5B*, *KDM5C*, and *KDM6A*. Overall, there were no significant associations between the expression of *KDM5D* and other genes of the KDM family at the TSS expression level (Figure 2D); however, there were significant inverse correlations in the expression of subsets of TSSs, including the higher expression of TSS region 1 of *KDM1A* (*r* = −0.610) and *KDM1*B (*r* = −0.557), and TSS region 3 of *KDM1B* (*r* = −0.828), in SCCs with *KDM5D* copy number loss (Figure 2E and supplementary material, Table S4).

### Clinicopathological characteristics of SCC with *KDM5D* copy number loss

**Figure 3:**
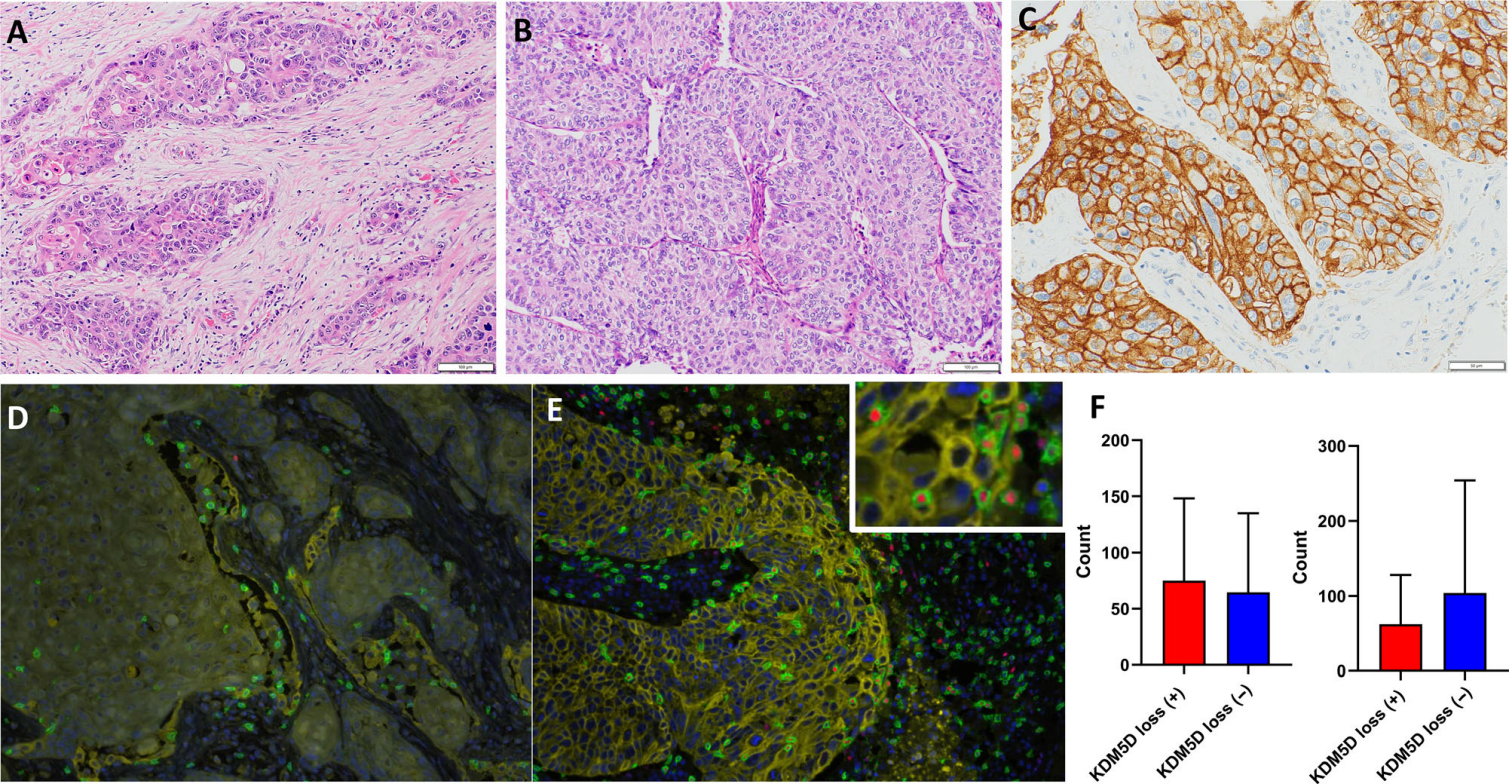
Clinicopathological characteristics of SCC with copy number loss of *KDM5D*. Tumor stromal content was categorized as (A) high (≥50% of the whole tumor area) and (B) low (<50%). (C) Representative image of high expression of PD‐L1 in SCC with *KDM5D* copy number loss. (D and E) Representative staining of multiplex immunohistochemistry of cytokeratin (yellow), CD8 (green), and T‐bet (red) in SCC (D) with and (E) without *KDM5D* copy number loss. (F) The number of infiltrating CD8^+^/T‐bet^+^ T cells in stroma was similar between SCC with *KDM5D* copy number loss and wild‐type tumors (left), whereas the number of infiltrating CD8^+^/T‐bet^+^ T cells within tumor in SCC with *KDM5D* copy number loss was lower than that in wild‐type tumors (right).

To explore the effects of clinicopathological characteristics of SCC with *KDM5D* copy number loss, the clinicopathological factors were evaluated in the 173 SCCs. The clinicopathological characteristics of 75 SCCs with *KDM5D* copy number loss are summarized in Table 1. SCCs with *KDM5D* copy number loss were significantly associated with lymph node metastasis (*p* = 0.0107) and late pathological stage (*p* = 0.0085). There was no significant correlation between copy number loss of *KDM5D* and other clinicopathological features such as age, smoking index, tumor size, pathological grade, lymphovascular invasion, *KRAS* mutation status, and PD‐L1 expression status. We further evaluated histological features in SCC with *KDM5D* copy number loss. SCCs with *KDM5D* copy number loss were significantly associated with high stromal content (*p* = 0.0254) (Figure 3A,B). There were no significant differences in other histological features including, nuclear area (supplementary material, Figure S1C), existence of tumor budding, and spread through alveolar spaces between tumors with *KDM5D* copy number loss and wild‐type tumors. High expression of PD‐L1 was identified in 23 (31%) patients with *KDM5D* copy number loss; however, a comparison between copy number loss of *KDM5D* and wild‐type tumors revealed no significant differences in PD‐L1 expression (Figure 3C). Multiplexed fluorescent immunohistochemistry analysis showed that CD8^+^/T‐bet^+^ T cells equally infiltrated the stroma of tumors with and without copy number loss of *KDM5D*. In contrast, CD8^+^/T‐bet^+^ T cells infiltrated tumors with *KDM5D* copy number loss less than wild‐type tumors, but these differences did not reach statistical significance (Figure 3D–F). The results of the transcriptome and multiplexed fluorescent immunohistochemistry analyses are summarized in supplementary material, Table S5.

### Clinical outcomes

**Figure 4:**
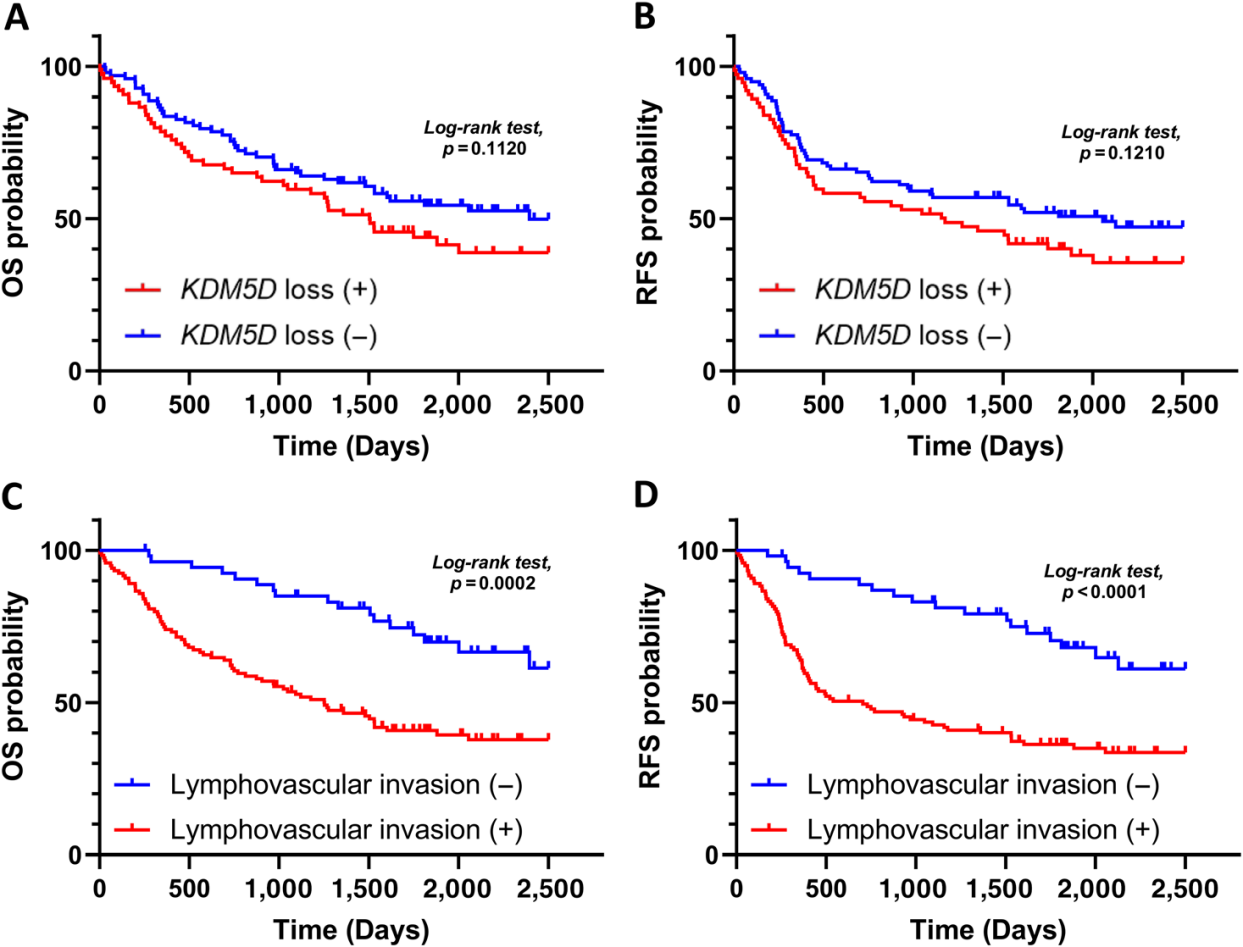
Kaplan–Meier curves of OS and RFS of patients with SCC harboring *KDM5D* copy number loss. OS and RFS according to the status of (A and B) *KDM5D* copy number and (C and D) existence of lymphovascular invasion.

With respect to clinical outcomes, the median follow‐up period after surgery for all patients was 1,482 days. In our cohort, the overall survival (OS) and recurrence‐free survival (RFS) rates were significantly associated with the pathological stage (OS, *p =* 0.0284; RFS, *p =* 0.0029) (supplementary material, Figure S3A,B). Patients with *KDM5D* copy number loss tended to have worse OS (*p =* 0.1220) and RFS rates (*p =* 0.1210), but these differences were not significant (Figure 4A,B). Regarding other clinicopathological parameters, the OS and RFS rates were significantly associated with lymphovascular invasion (OS, *p =* 0.0002; RFS, *p <* 0.0001) in patients with SCC (Figure 4C,D), whereas there were no significant differences in histological types (supplementary material, Figure S3C,D) and expression level of PD‐L1 (supplementary material, Figure S3E,F). In this cohort, 51 patients developed recurrence. Among them, five patients were treated with immune checkpoint inhibitors (ICIs): four patients died of disease (two patients with *KDM5D* copy number loss and low expression of PD‐L1; one patient with *KDM5D* copy number loss and high expression of PD‐L1; one patient with *KDM5D* wild type and low expression of PD‐L1) and one patient with *KDM5D* copy number loss and high expression of PD‐L1 was alive with disease (supplementary material, Figure S4).

## Discussion

Enrichment of repetitive and palindromic sequences on the Y chromosome makes bioinformatic studies of copy number alterations on the Y chromosome challenging [15]. Thus, in the present study we used FISH, the gold standard for detecting copy number changes in tumor cells, and successfully determined that approximately 40% of the patients had *KDM5D* copy number loss in SCC. Furthermore, the transcriptome analysis results indicated that the expression of TSS region 1 of *KDM5D* was reduced in SCCs with *KDM5D* copy number loss, suggesting that copy number loss of *KDM5D* could lead to the absence of *KDM5D* transcripts and the subsequent loss of KDM5D protein expression in SCC. Reduced expression of *KDM5D* in human cancer cells has been reported in various organs such as the prostate [15], kidney [32], stomach [33], and lung [34]. Notably, the downregulation of *KDM5D* induces an aggressive phenotype in prostate and gastric carcinomas [15, 35]. Male patients with *KDM5D* deficiency in NSCLC tumors show a substantially increased risk of death [34]. These findings are consistent with our clinicopathological results, showing patients with *KDM5D* copy number loss tended to exhibit worse OS. Histologically, stromal content was exclusively different in SCCs with and without *KDM5D* copy number loss, and there were no apparent aggressive histological features in SCC with *KDM5D* copy number loss. This suggests that SCC with *KDM5D* copy number may be characterized by its unique tumor microenvironment (TME). Moreover, activated ATR signaling with the loss of *KDM5D* expression can be exploited to elicit synthetic lethality of ATR inhibition in cancer cells [15]. The discovery of clinically targetable genomic alterations in NSCLC has revolutionized the treatment of patients with NSCLCs. Furthermore, such patient stratification has started to permeate therapeutic strategies for SCLCs based on the expression of transcription factors including ASCL1, NEUROD1, and POU2F3 [36]. However, ongoing clinical trials to advance the development of targeted therapies for SCC are limited. Thus, patients with SCCs harboring copy number loss of *KDM5D* may be candidates for treatment with ATR inhibitors.

Our transcriptome data demonstrated that TSSs of genes on the Y chromosome around *KDM5D* were significantly lower in tumors with *KDM5D* copy number loss than wild‐type tumors, implying that somatic loss of the Y chromosome including the *KDM5D* locus may occur in tumors with *KDM5D* copy number loss. Similarly, a previous study revealed a deficiency of *KDM5D* with low expression of the other eight Y chromosome transcripts because of a partial somatic deletion in NSCLC [34]. Consequently, our findings may be affected by not only the copy number loss of *KDM5D*, but also aberration in other genes on the Y chromosome; however, such genes play a limited role in carcinogenesis [37]. Alternatively, SCCs with *KDM5D* copy number loss exhibited high expression of TSS region 1 of *KDM1A*. Thus, the interplay of *KDM5D* and *KDM1A* may have resulted in the clinicopathological findings of the present study, because the final effect on chromatin structure and genome function in most cancers is not strictly dependent on one histone modification [38].

Our multiplex immunohistochemical results along with transcriptome data indicate that SCCs with copy number loss of *KDM5D* can be ‘cold tumors’, which are characterized by the paucity of tumor T‐cell infiltration and usually do not respond to immunotherapy. This observation is inconsistent with that of previous studies, which showed that dysfunction of the DNA damage response pathway in tumor cells is capable of activating an immune response possibly by increasing the level of neoantigen and tumor immunogenicity [39, 40]. Currently, the molecular mechanism underlying the paucity of tumor T‐cell infiltration within tumors and subsequent downregulation of genes associated with the immune response in SCCs with copy number loss of *KDM5D* is unclear. However, loss‐of‐function *RNF45* mutations reduce CC chemokine ligand (CCL4) levels and prevent CD8^+^ T cell infiltrations, leading to noninflamed TME in high‐frequency microsatellite instability colorectal adenocarcinomas [41]. Furthermore, some of the neoantigenic mutations, particularly driver mutations, can paradoxically act as antitumor immunity suppressors through the original gene function [41]. Such ‘paradoxical neoantigenic mutations’ may occur in SCCs with *KDM5D* copy number loss. Alternatively, our results indicate that the histology of stroma in SCCs with and without *KDM5D* copy number loss is different, suggesting that unique cellular constituents within the TME in SCCs with *KDM5D* copy number loss affect the distinct immunological attributes. Further studies with other approaches such as spatial transcriptomics will be needed to elucidate the regulation of the TME in SCCs with *KDM5D* copy number loss.

A limitation of this study is that we detected *KDM5D* copy number loss in male patients with SCC using FISH. This method has been successfully used to identify prostate adenocarcinoma with *KDM5D* copy number loss [15]. However, the association of copy number loss of *KDM5D* and loss of protein expression of KDM5D using immunohistochemistry was not evaluated in FFPE specimens. The immunohistochemical analysis could be useful in clinical settings to select patients for this new targeted therapy. Therefore, development of a KDM5D‐specific antibody is needed. Finally, only five patients with *KDM5D* copy number loss were treated with ICI in the present study. Thus, it is still unclear whether patients with *KDM5D* copy number loss show positive or negative responses to treatment with ICI. However, predictive markers of ICI efficacy have been gradually explored from the expression of intermolecular interactions within tumor cells to the expression of various molecules and cells in the TME, and have been extended to the exploration of circulating and host systemic markers [42]. Thus, further clinical analyses using multifactorial synergistic predictive markers are needed to determine the response to ICI in patients with *KDM5D* copy number loss and patient stratification for determining optimal therapy for lung cancer.

In summary, FISH‐based *KDM5D* screening revealed that approximately 40% of male patients with SCC have copy number loss of *KDM5D*. Although additional studies are required to clarify the clinicopathological effects of *KDM5D* loss in SCC through external validation, patients whose tumors show this distinct phenotype, especially in a subgroup of patients without PD‐L1 expression, could benefit from targeted therapies involving ATR inhibitors.

## Author contributions statement

THay and KK conceived and designed the study. AU, KK, ES, SS and SW developed the methodology for the study. AU, THay, KK, MH, ES, SS, SW, THan, TS, KT, SK, KS and TY were in charge of acquisition of the data. AU, THay, KK, MH, ES, SS, SW, THan, TS, KT, SK, KS and TY analyzed and interpreted the data. All authors were involved in writing the paper and provided final approval of the submitted and published versions.

## Supporting information


**Figure S1.** The QuPath‐based method is applicable for analysis of cell area measurement
**Figure S2.** Correlation analysis between FISH and ddPCR
**Figure S3.** Kaplan–Meier curves of OS and RFS
**Figure S4.** The timeline of progression of patients who were treated with immune checkpoint inhibitors

**Table S1.** Clinicopathological features and results of ddPCR and FISH in patients with small cell carcinoma

**Table S2.** TSS‐level transcriptome expression in lung squamous cell carcinoma

**Table S3.** Differentially expressed TSSs between squamous cell carcinoma with and without *KDM5D* copy number loss

**Table S4.** TSS‐level expression profiles of the lysine demethylases in tumors with and without *KDM5D* copy number loss

**Table S5.** Summary of experimental results in lung squamous cell carcinoma with *KDM5D* copy number loss

## Data Availability

The data that support the findings of this study are available on request from the corresponding author. The data are not publicly available due to privacy and ethical restrictions.
