# Supplementary material for: Copy number loss of KDM5D may be a predictive biomarker for ATR inhibitor treatment in male patients with pulmonary squamous cell carcinoma
Source: J Pathol Clin Res. 2023 Nov 16;10(1):e350. doi: 10.1002/cjp2.350 (PMC10766025; doi:10.1002/cjp2.350)
Supplement: Supplementary file 1 — Figure S1. The QuPath‐based method is applicable for analysis of cell area measurement Figure S2. Correlation analysis between FISH and ddPCR Figure S3. Kaplan–Meier curves of OS and RFS Figure S4. The timeline of progression of patients who were treated with immune checkpoint inhibitors [file CJP2-10-e350-s005.pdf]

Copy number loss of *KDM5D* may be a predictive biomarker for ATR inhibitor treatment in male patients with pulmonary squamous cell carcinoma

A Ura *et al*., *J Pathol Clin Res,* https://doi.org/10.1002/cjp2.350

Supplementary Figures S1–S4

Supplementary Tables S1–S5 (see separate Excel files)

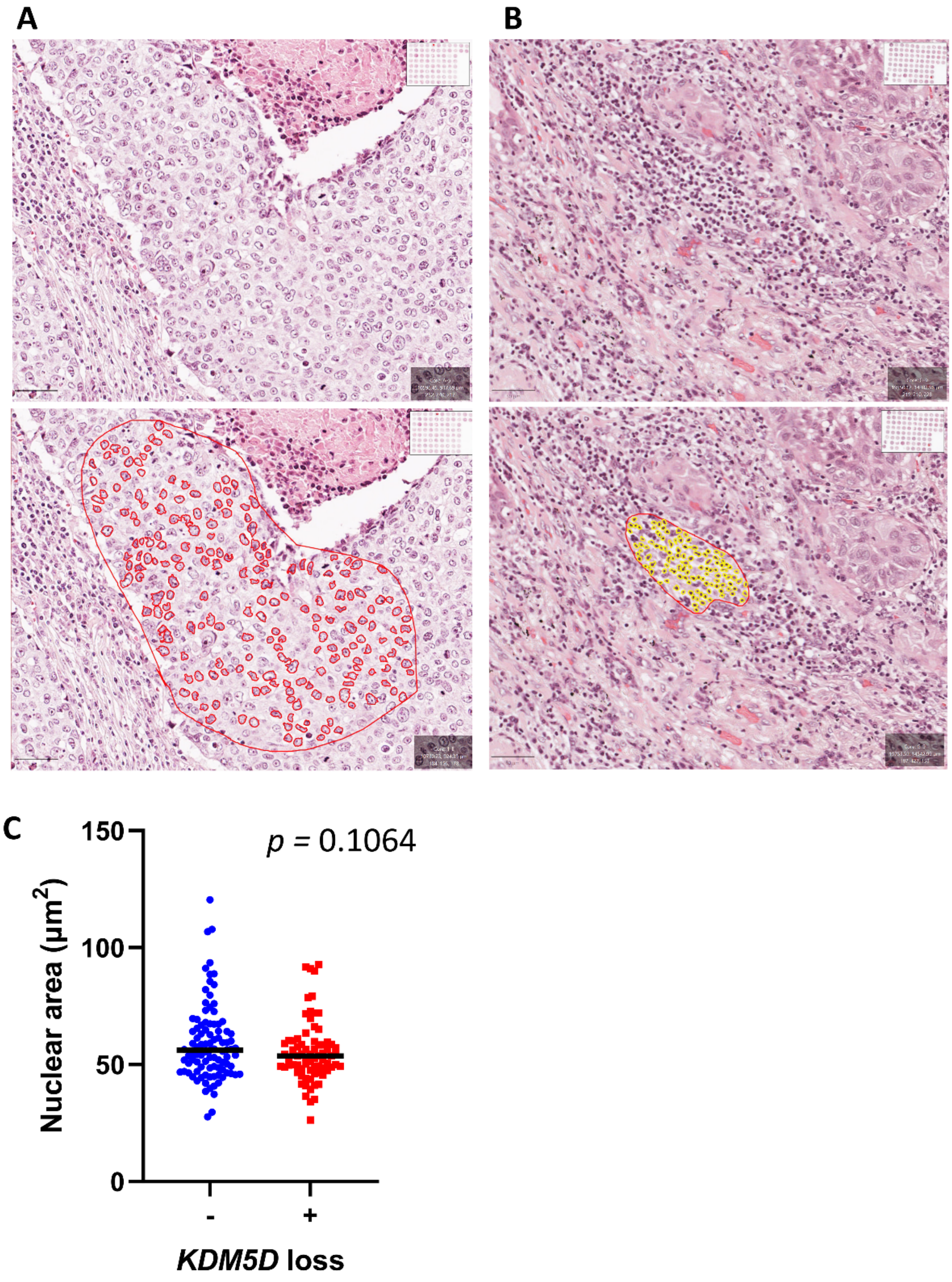

Figure S1. The QuPath-based method is applicable for analysis of cell area measurement. **(A)** Representative images of annotating tumor cells of interest using Qupath's polygon tool, original magnification ×200, and scale bar 50 μm.

(B) Representative images of annotating lymphocytes by using Qupath's polygon tool, original magnification ×200, and scale bar 50 μm. Median nuclear area in tumor cells is 54.28 μm^2 (range: 26.30–120.50 μm^2). As a control, mean nuclear area in lymphocytes was 18.5 μm^2 (range: 5.27–42.19 μm^2). **(C)** There is no significant correlation between copy number loss of *KDM5D* and nuclear area.

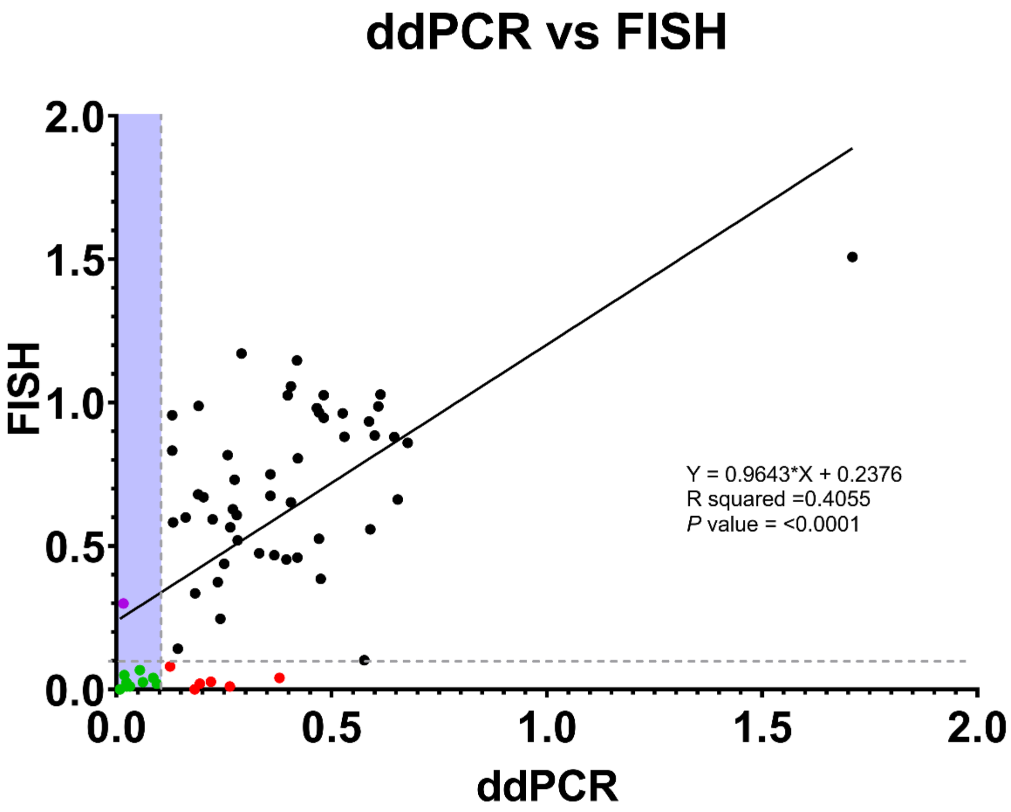

Figure S2. Correlation analysis between FISH and ddPCR. Loss of *KDM5D* detected using FISH correlated with that of ddPCR in a control set of 65 small cell lung carcinomas (p < 0.0001). The x-axis represents *KDM5D/SPIN4* ratio in ddPCR, while the y-axis represents *KDM5D/CenX* ratio in FISH

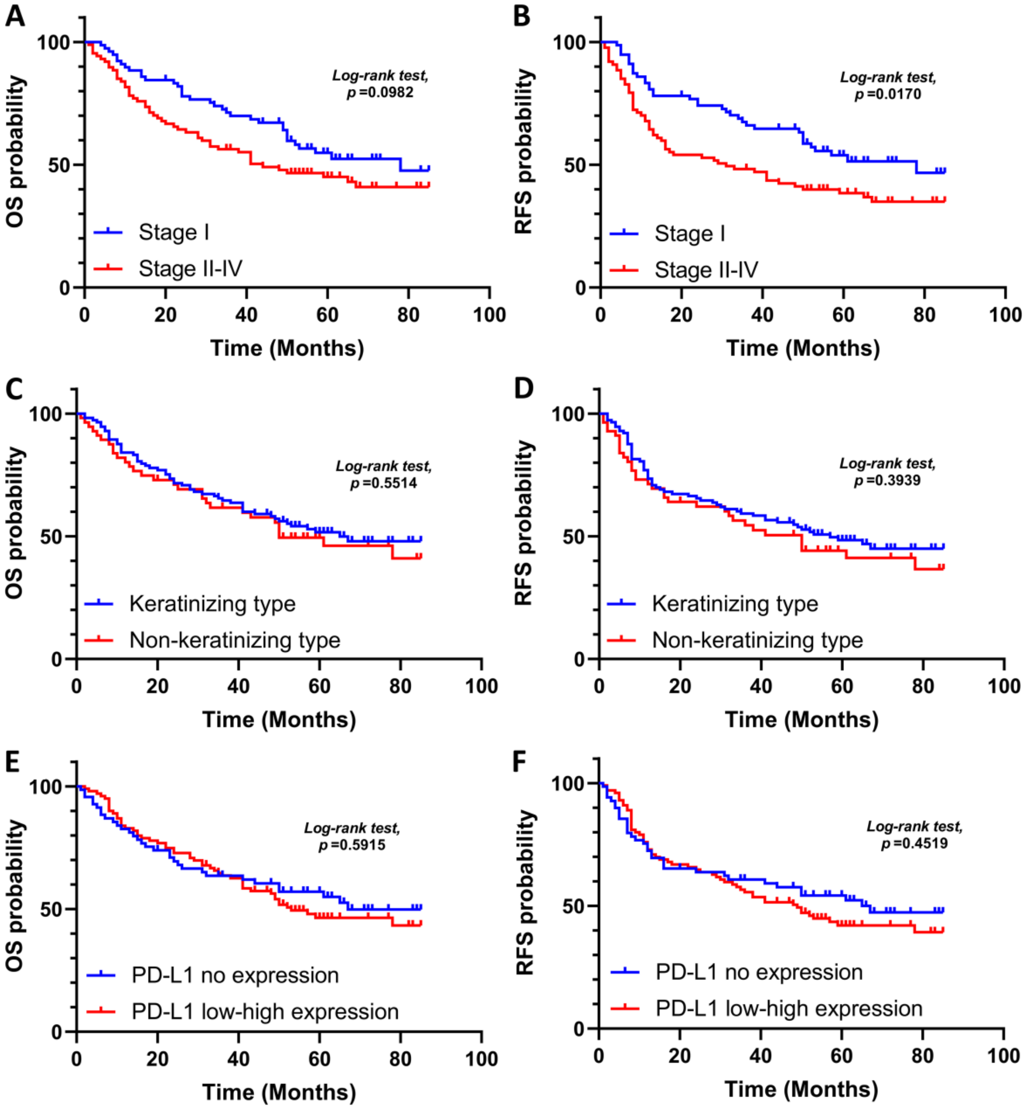

Figure S3. Kaplan–Meier curves of overall survival (OS) and recurrence-free survival (RFS). OS and RFS according to **(A,B)** pathological stage, **(C,D)** histological type, and **(E,F)** PD-L1 expression.

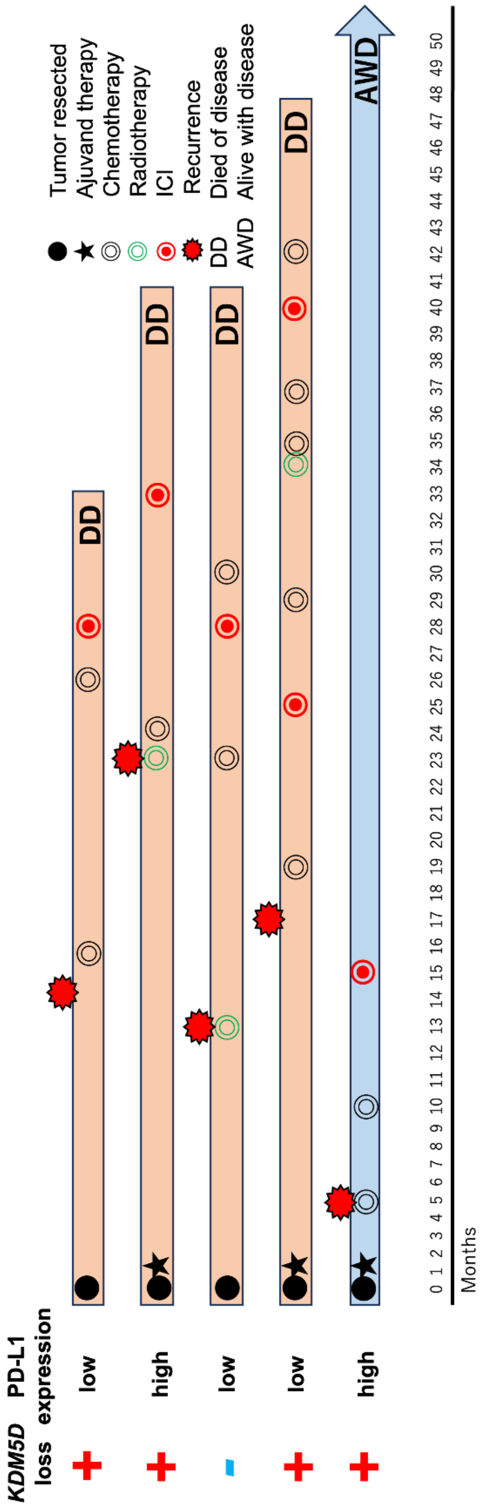

Figure S4. The timeline of progression of patients who were treated with immune checkpoint inhibitors (ICI).
